# Improving Generalizability of Clinical Research by Enrolling Low Socioeconomic Status Participants from Federally Qualified Health Care Centers

**DOI:** 10.1002/alz70860_099542

**Published:** 2025-12-23

**Authors:** Susan M. Landau, Melanie J. Miller, Catherine C. Conti, Juliet Fockler, Diana Truran‐Sacrey, Adam Diaz, Miriam T. Ashford, Rachel L. Nosheny, Ozioma C. Okonkwo, Monica G Rivera Mindt, Michael W Weiner, Alzheimer's Disease Neuroimaging Initiative

**Affiliations:** ^1^ Neuroscience Department, University of California, Berkeley, Berkeley, CA, USA; ^2^ Northern California Institute for Research and Education (NCIRE), San Francisco, CA, USA; ^3^ University of California, San Francisco, San Francisco, CA, USA; ^4^ Center for Health Disparities Research, Department of Medicine, School of Medicine and Public Health (SMPH), University of Wisconsin‐Madison, Madison, WI, USA; ^5^ Icahn School of Medicine, Mount Sinai Hospital, New York, NY, USA; ^6^ Fordham University, New York, NY, USA; ^7^ Northern California Institute for Research & Education (NCIRE), San Francisco, CA, USA

## Abstract

**Background:**

A major limitation of the Alzheimer's Disease Neuroimaging Initiative (ADNI) and most ADRD clinical research is lack of generalizability due to under‐inclusion of low socioeconomic status (LSES) participants. ADNI is planning a sub‐study to explore methods and determine feasibility of engaging and enrolling LSES participants.

**Method:**

ADNI plans to use two approaches to identify, engage, enroll LSES participants: (1) Several organizations have large databases with health care and SES indicators (e.g. education, occupation, income) on millions of individuals. These databases can identify LSES individuals in various geographic areas and provide contact information for informational marketing and recruitment efforts. (2) Thousands of Federally Qualified Health Clinics (FQHCs) across the USA provide primary care to LSES communities. Although several networks of FQHCs have enrolled LSES participants in clinical research, to our knowledge such individuals have not been previously included in AD biomarker research. ADNI plans to use these approaches to enroll 100‐200 LSES participants in a sub‐study. ADNI leadership and staff will extensively interact with relevant organizations and members of the FQHC community to understand the needs of clients and clinic staff, and to optimize the engagement and enrollment approach. To enroll LSES participants from diverse geographical regions, including rural areas, participants may be assessed at existing ADNI sites or new assessment sites using remote assessment.

**Result:**

Participants will be assessed with a simplified ADNI battery: clinical measures (Clinical Dementia Rating, brief tests of memory and executive function), routine blood screening (HbA1C, cholesterol) and AD plasma biomarkers. A subset will undergo neuroimaging (MRI, amyloid PET, tau PET). Critically, acquisition and analysis of all measurements will be harmonized with existing ADNI procedures to enable direct comparison with decades of legacy ADNI measurements. The first participants are expected to be enrolled in late 2025.

**Conclusion:**

This sub‐study will identify the most effective methods to engage and enroll LSES participants into ADRD biomarker research. These methods will also evaluate feasibility for ADNI5, which aims to establish a national cohort of participants over age 55 who approximate the US census for gender, race, ethnicity, socioeconomic status and geographic distribution.

